# Chitosan (CTS) Alleviates Heat-Induced Leaf Senescence in Creeping Bentgrass by Regulating Chlorophyll Metabolism, Antioxidant Defense, and the Heat Shock Pathway

**DOI:** 10.3390/molecules26175337

**Published:** 2021-09-02

**Authors:** Cheng Huang, Yulong Tian, Bingbing Zhang, Muhammad Jawad Hassan, Zhou Li, Yongqun Zhu

**Affiliations:** 1 College of Grassland Science and Technology, Sichuan Agricultural University, Chengdu 611130, China; gavincheng@stu.sicau.edu.cn (C.H.); cjdsb20001029tyl@163.com (Y.T.); fadedbing@163.com (B.Z.); jawadhassan3146@gmail.com (M.J.H.); 2 Soil and Fertilizer Research Institute, Sichuan Academy of Agricultural Sciences, Chengdu 610066, China

**Keywords:** photochemical efficiency, water balance, heat shock protein, heat shock factor, gene expression, thermotolerance, heat stress

## Abstract

Chitosan (CTS) is a deacetylated derivative of chitin that is involved in adaptive response to abiotic stresses. However, the regulatory role of CTS in heat tolerance is still not fully understood in plants, especially in grass species. The aim of this study was to investigate whether the CTS could reduce heat-induced senescence and damage to creeping bentgrass associated with alterations in antioxidant defense, chlorophyll (Chl) metabolism, and the heat shock pathway. Plants were pretreated exogenously with or without CTS (0.1 g L^−1^) before being exposed to normal (23/18 °C) or high-temperature (38/33 °C) conditions for 15 days. Heat stress induced detrimental effects, including declines in leaf relative water content and photochemical efficiency, but significantly increased reactive oxygen species (ROS) accumulation, membrane lipid peroxidation, and Chl loss in leaves. The exogenous application of CTS significantly alleviated heat-induced damage in creeping bentgrass leaves by ameliorating water balance, ROS scavenging, the maintenance of Chl metabolism, and photosynthesis. Compared to untreated plants under heat stress, CTS-treated creeping bentgrass exhibited a significantly higher transcription level of genes involved in Chl biosynthesis (*AsPBGD* and *AsCHLH*), as well as a lower expression level of Chl degradation-related gene (*AsPPH*) and senescence-associated genes (*AsSAG12*, *AsSAG39*, *Asl20*, and *Ash36*), thus reducing leaf senescence and enhancing photosynthetic performance under heat stress. In addition, the foliar application of CTS significantly improved antioxidant enzyme activities (SOD, CAT, POD, and APX), thereby effectively reducing heat-induced oxidative damage. Furthermore, heat tolerance regulated by the CTS in creeping bentgrass was also associated with the heat shock pathway, since *AsHSFA-6a* and *AsHSP82* were significantly up-regulated by the CTS during heat stress. The potential mechanisms of CTS-regulated thermotolerance associated with other metabolic pathways still need to be further studied in grass species.

## 1. Introduction

High temperatures are one of the most critical environmental factors affecting plant growth and geographical distribution [1]. In recent years, the enormous greenhouse gas emissions resulting from anthropogenic activities have led to a yearly increase in global temperatures, which has drastically influenced the growth and yield of economical plants worldwide. Between 1980 and 2008, the increase in the global average temperature has caused a 5.5% decrease in wheat (*Triticum aestivum*) production [2]. Heat stress also adversely affects turfgrass growth and management, leading to a significant decrease in turf quality during summer as well as an increase in lawn maintenance costs [3,4]. The exogenous application of chemical compounds can effectively improve the tolerance of plants to various abiotic stresses such as drought, salinity, and high temperatures. For example, the exogenous application of spermidine and sucrose could significantly improve tolerance to heat stress in rice (*Oryza sativa*), tomato (*Lycopersicon esculentum*), and potato (*Solanum tuberosum*) by enhancing photosynthesis and antioxidant capacity [5,6,7]. The application of melatonin could effectively alleviate the adverse effects of salt stress on maize (*Zea mays*) [8], drought stress on creeping bentgrass (*Agrostis stolonifera*) [9], and heat stress on perennial ryegrass (*Lolium perenne*) [10]. Heat tolerance could be improved significantly by exogenous methyl jasmonate in perennial ryegrass associated with the altered osmotic adjustment, antioxidant defense, and transcript levels of jasmonate-responsive genes [11].

Chitosan (CTS), a deacetylation product of chitin, is one of the most abundant natural polysaccharides in nature [12]. Due to its non-toxicity, cheap price, biocompatibility, and biodegradable properties, CTS has been widely used in agriculture and biotechnology [13]. CTS is also considered to be a promising exogenous additive for the improvement of crop yield and tolerance of abiotic stresses. It has been shown that CTS enhances leaf membrane stability and antioxidant enzyme activities in apple (*Malus domestica*) seedlings under drought stress [14]. Exogenous CTS could improve the salt tolerance of sweet pepper (*Capsicum annuum*) by affecting chlorophyll fluorescence parameters and antioxidant defense [15]. The maintenance of better plant growth and photosynthetic pigments could be achieved by the exogenous application of CTS in rice suffering from osmotic stress [16]. Our previous studies have also found that CTS application is an effective approach to regulate the drought tolerances of white clover (*Trifolium repens*) and creeping bentgrass [17,18]. The study of Ibrahim and Ramadan found that the foliar spray of a combination of zinc and CTS could alleviate heat-induced growth inhibition and malnutrition in dry bean (*Phaseolus vulgaris*) [19]. However, the regulatory mechanism of CTS on heat tolerance in plants (especially grass species) has so far remained unclear.

Creeping bentgrass is an important member of the *graminae* family, which is used worldwide on golf courses, green tennis courts, and landscapes because of its fast establishment rate, excellent texture, and ability to form beautiful lawns [20]. However, as a cool season turfgrass, its low heat tolerance is often the major factor limiting its cultivation and utilization in most countries and regions with summer temperatures above 30 °C [21]. In this study, the ameliorative effect of foliar CTS application on the heat tolerance of creeping bentgrass was investigated based on various physiological analyses. In addition, osmotic adjustment, antioxidant defense, the heat shock pathway, and heat-induced leaf senescence associated with changes in chlorophyll metabolism were further examined to reveal the possible mechanisms of thermotolerance induced by CTS in creeping bentgrass.

## 2. Results

### 2.1. Effects of the CTS on Water Status, Photochemical Efficiency, and Chl Metabolism

**Figure 1:**
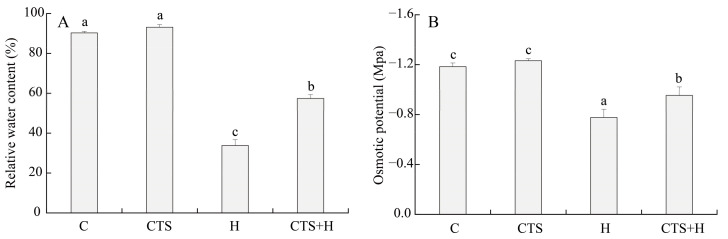
Effect of chitosan on (**A**) relative water content (RWC) and (**B**) osmotic potential (OP) under control and heat stress conditions. Vertical bars above columns indicate ± standard error (SE) of means (*n* = 4) and different letters above columns indicate significant differences among treatments (*p* ≤ 0.05).

**Figure 2:**
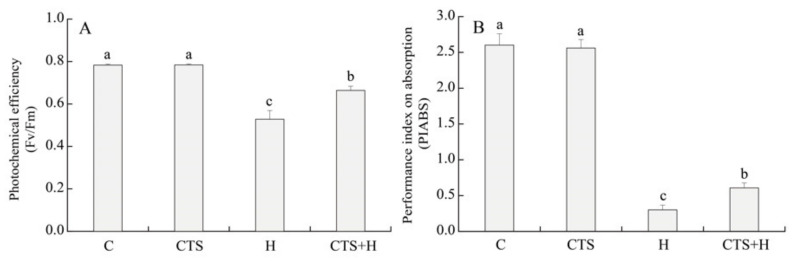
Effect of chitosan on (**A**) photochemical efficiency (Fv/Fm) and (**B**) performance index on absorption basis (PIABS) under control and heat stress conditions. Vertical bars above columns indicate ± standard error (SE) of means (*n* = 4) and different letters above columns indicate significant differences among treatments (*p* ≤ 0.05).

Exogenous CTS had no significant impacts on relative water content (RWC) and osmotic potential (OP) in leaves under normal conditions (Figure 1A,B). Compared to the control, the RWC significantly decreased under heat stress in creeping bentgrass treated with or without CTS. Results showed that CTS-treated plants exhibited a significantly higher RWC than untreated plants under heat stress (Figure 1A). In addition, heat stress significantly increased OP in both CTS-treated and untreated plants, but the CTS-treated creeping bentgrass had a lower OP than the untreated plants (Figure 1B). Exogenous CTS did not significantly affect photochemical efficiency (Fv/Fm) or performance index on absorption basis (PIABS) in creeping bentgrass under normal conditions (Figure 2A,B). Heat stress significantly decreased Fv/Fm and PIABS, but CTS-treated plants had significantly higher Fv/Fm and PIABS than untreated plants (Figure 2A,B).

**Figure 3:**
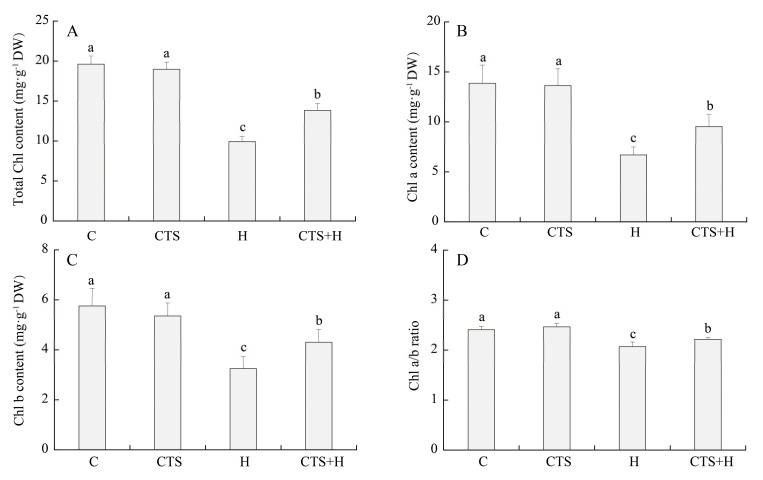
Effect of chitosan on (**A**) total chlorophyll (Chl) content, (**B**) Chl a content, (**C**) Chl b content, and (**D**) Chl a/b ratio under control and heat stress conditions. Vertical bars above columns indicate ± standard error (SE) of means (*n* = 4) and different letters above columns indicate significant differences among treatments (*p* ≤ 0.05).

**Figure 4:**
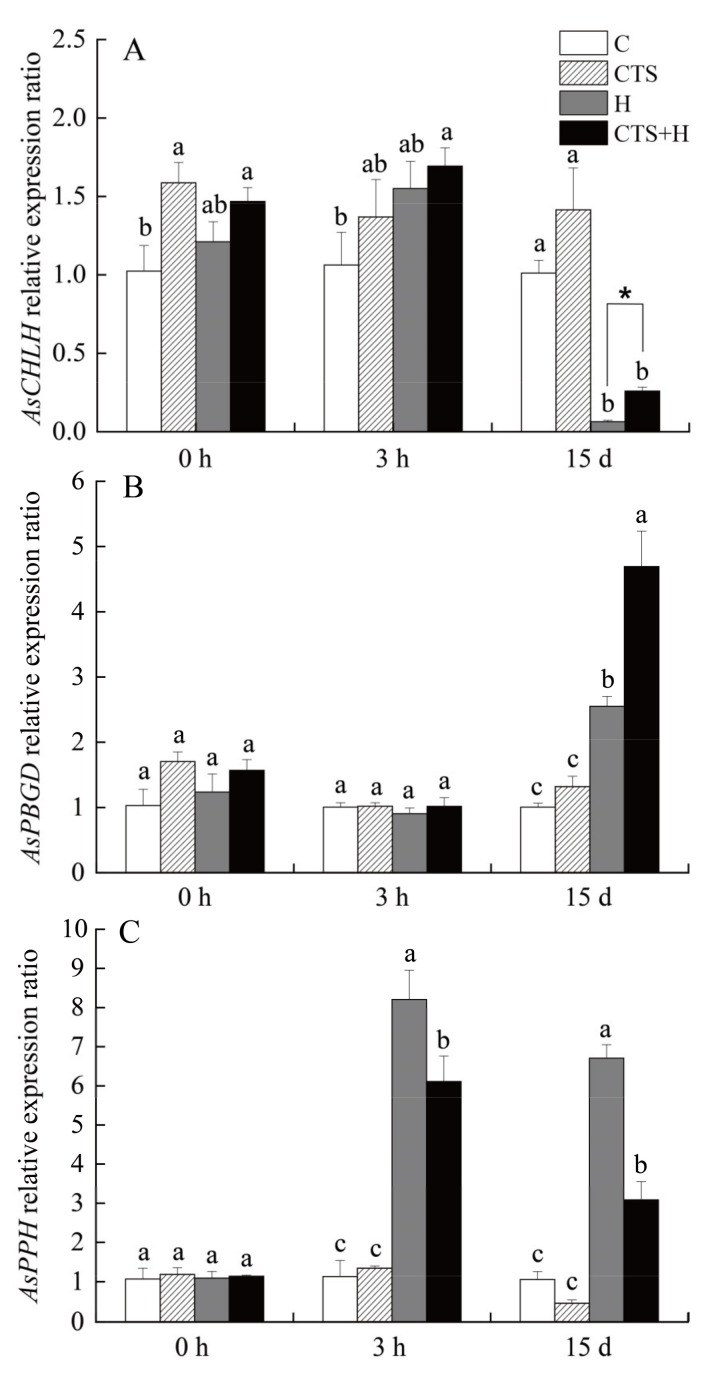
Effect of chitosan on (**A**) *AsCHLH*, (**B**) *AsPBGD*, and (**C**) *AsPPH* relative expression in leaves of creeping bentgrass under control and heat stress conditions. Vertical bars above columns indicate ± standard error (SE) of means (*n* = 4) and different letters above columns indicate significant differences among treatments at one particular time point (*p* ≤ 0.05).

Heat stress (15 days) induced a significant decrease in the content of total Chl, Chl a, and Chl b, as well as in the Chl a/b ratio, but these parameters remained at significantly higher levels in CTS-treated plants (Figure 3A–D). As shown in Figure 4, exogenous CTS significantly increased the transcript levels of *AsCHLH* and *AsPBGD* but exhibited no significant effect on the transcript level of *AsPPH* at 0 h. Heat stress significantly inhibited *AsCHLH* expression but increased *AsPBGD* expression level in both CTS-pretreated and untreated plants (Figure 4A,B). On the 15th day of heat stress, the transcript levels of *AsCHLH* and *AsPBGD* in CTS-treated plants were 4.01 times and 1.84 times higher, respectively, than those in untreated plants (Figure 4A,B). Heat stress greatly enhanced the transcript level of *AsPPH* in both CTS-treated and untreated creeping bentgrasses. However, the *AsPPH* expression levels in CTS-treated plants showed a 25.5% and 54.0% decrease, respectively, compared to those of untreated plants at the 3 h or 15 d of heat stress (Figure 4C).

### 2.2. Effects of the CTS on Oxidative Damage and Antioxidant Enzyme Activities

**Figure 5:**
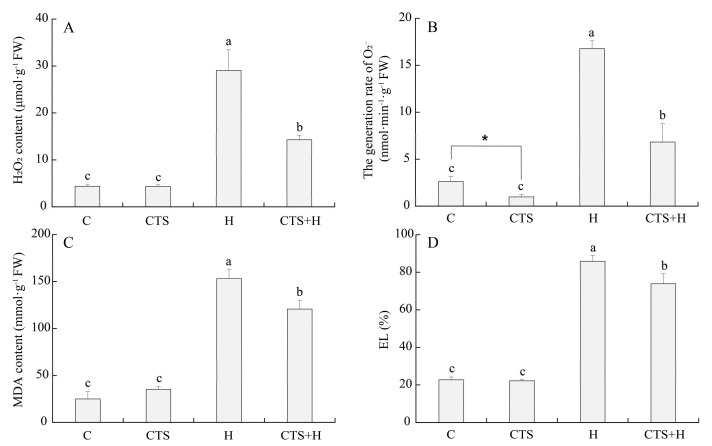
Effect of chitosan on (**A**) hydrogen peroxide (H_2_O_2_) content, (**B**) superoxide anion (O_2_^–^), (**C**) malondialdehyde (MDA) content, and (**D**) electrolyte leakage (EL) under control and heat stress conditions. Vertical bars above columns indicate ± standard error (SE) of means (*n* = 4) and different letters above columns indicate significant differences among treatments (*p* ≤ 0.05).

**Figure 6:**
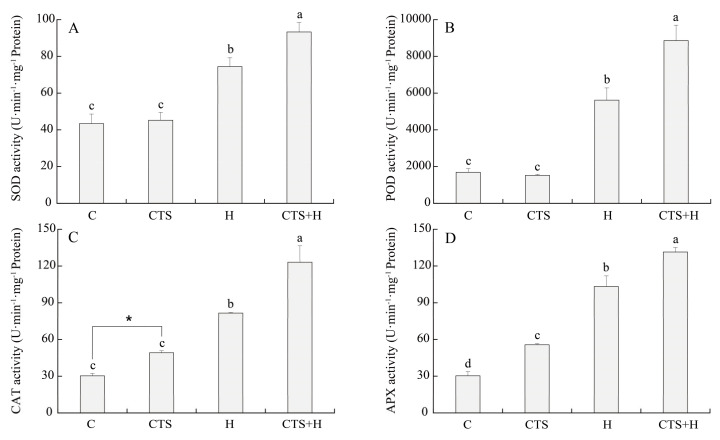
Effect of chitosan on (**A**) superoxide dismutase (SOD), (**B**) peroxidase (POD), (**C**) catalase (CAT), and (**D**) ascorbate peroxidase (APX) activities under control and heat stress conditions. Vertical bars above columns indicate ± standard error (SE) of means (*n* = 4) and different letters above columns indicate significant differences among treatments (*p* ≤ 0.05).

Although the hydrogen peroxide (H_2_O_2_) content, malondialdehyde (MDA) content, and electrolyte leakage (EL) were not significantly affected by the exogenous application of CTS under normal conditions, the use of CTS significantly reduced the generation of superoxide anion (O_2_^–^) under non-stress conditions (Figure 5A–D). Heat stress caused significant increases in H_2_O_2_, O_2_^–^, MDA, and EL in the leaves of CTS-treated and untreated plants. However, the exogenous application of CTS significantly decreased the H_2_O_2_, O_2_^–^, MDA, and EL by 50.8%, 59.3%, 21.2%, and 13.9%, respectively, when compared with untreated plants under heat stress (Figure 5A–D). Exogenous CTS also significantly increased the catalase (CAT) and ascorbate peroxidase (APX) activities, but had no significant effect on the superoxide dismutase (SOD) and peroxidase (POD) activities under normal conditions (Figure 6). Heat stress significantly increased the activities of SOD, POD, CAT, and APX in the leaves of the CTS-untreated creeping bentgrass. These antioxidant enzyme activities were further enhanced by exogenous CTS under heat stress. The SOD, POD, CAT, and APX activities in CTS-treated plants increased by 25.3%, 57.8%, 60.0%, and 27.3%, respectively, compared to untreated plants under heat stress (Figure 6).

### 2.3. Effects of the CTS on Expression Level of Genes Involved in HSF Pathway and Senescence

**Figure 7:**
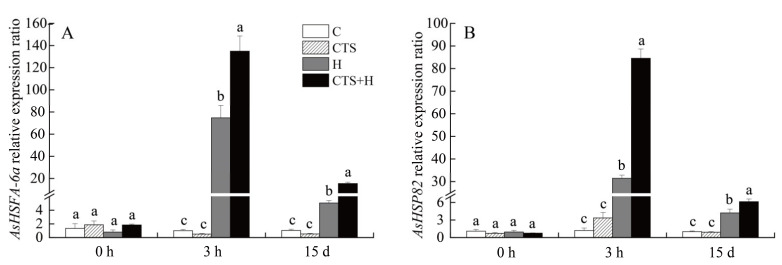
Effect of chitosan on the relative expression levels of (**A**) *AsHSFA-6a* and (**B**) *AsHSP82* under control and heat stress conditions. Vertical bars above columns indicate ± standard error (SE) of means (*n* = 4) and different letters above columns indicate significant differences among treatments at one particular time point (*p* ≤ 0.05).

**Figure 8:**
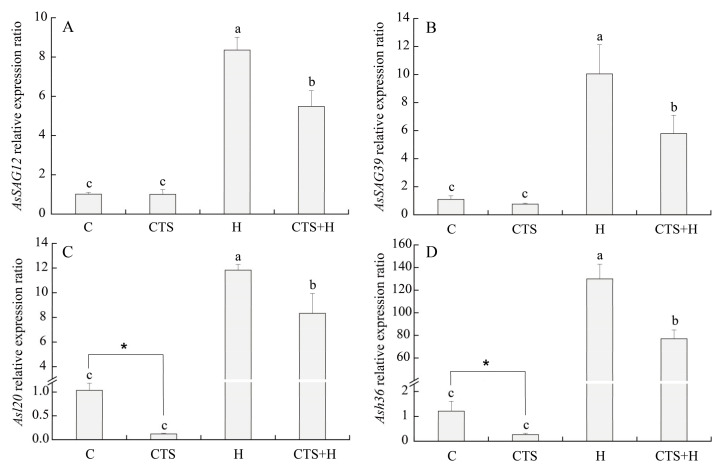
Effect of chitosan on the relative expression levels of (**A**) *AsSAG12*, (**B**) *AsSAG39*, (**C**) *Asl20*, and (**D**) *Ash36* under control and heat stress conditions. Vertical bars above columns indicate ± standard error (SE) of means (*n* = 4) and different letters above columns indicate significant differences among treatments (*p* ≤ 0.05).

**Figure 9:**
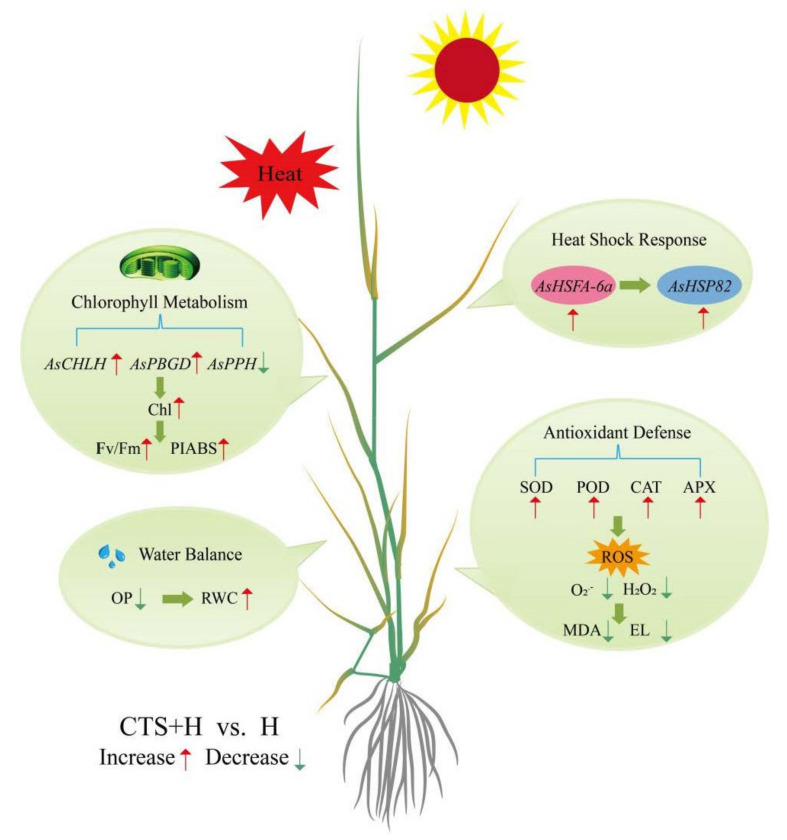
Integrative pathways regulated by chitosan in the leaves of creeping bentgrass under heat stress.

Exogenous CTS exhibited no significant effects on the transcription of *AsHSFA-6a* and *AsHSP82* under normal conditions (Figure 7). Heat stress significantly up-regulated the expression levels of *AsHSFA-6a* and *AsHSP82* at 3 h and 15 d; however, the peak value was observed at 3 h (Figure 7A,B). The transcript levels of *AsHSFA-6a* or *AsHSP82* in CTS-treated plants were 1.8 times and 2.7 times higher, respectively, than those of the untreated plants at 3 h of heat stress. After 15 days of heat stress, the transcript levels of *AsHSFA-6a* and *AsHSP82* in leaves of CTS-treated plants exhibited a 204.8% and 45.5% increase, respectively, compared to those in untreated plants (Figure 7A,B). Heat stress significantly up-regulated the expression of senescence-associated genes (*AsSAG12*, *AsSAG39*, *Asl20*, and *Ash36*) in CTS-treated and untreated creeping bentgrass, but the application of CTS significantly reduced the heat-induced increases in the expression levels of these genes (Figure 8A–D). Compared with the untreated plants under heat stress, the transcription of *AsSAG12*, *AsSAG39*, *Asl20*, or *Ash36* in CTS-treated plants decreased by 34.4%, 42.3%, 29.6%, or 40.7%, respectively (Figure 8A–D). Integrative pathways regulated by CTS in the leaves of creeping bentgrass under heat stress are shown in Figure 9.

## 3. Discussion

Heat stress causes a disruption in normal metabolic processes and damage cell membrane stability and photosynthetic apparatus, resulting in increased leaf wilting and senescence [22,23]. CTS is a natural biopolymer that is highly involved in a plant’s response to abiotic stresses via secondary messenger pathways [24]. Previous studies found that cowpea (*Vigna unguiculata*) leaves sprayed with 250 mg/L of CTS promoted Chl content and carbohydrate accumulation under drought stress [25]. Salt- or drought-induced water loss in leaves could be effectively alleviated by the exogenous application of CTS, which is associated with the enhancement of sugar accumulation and metabolism for improved osmotic adjustment (OA) in leaves of white clover and creeping bentgrass [17,18,26]. The maintenance of better photosynthetic capacity could be one of the most important factors for CTS-regulated sugar production and metabolism in creeping bentgrass under salt and drought stress [18,26]. Similar results were found in this study. Persistent high temperatures (38/33 °C, day/night) resulted in water loss and declines in the OA capacity of the CTS-treated and untreated creeping bentgrass. However, exogenous CTS pretreatment significantly prevented decreases in RWC, Fv/Fm, and PIABS induced by heat stress in leaves. This suggested that CTS exhibited a positive regulatory effect on water homeostasis and photosynthetic response when creeping bentgrass was subjected to a long period (15 days) of heat stress.

Chl is the main photosynthetic pigment in chloroplasts that performs light energy absorption and conversion. When plants are subjected to abiotic stresses such as heat stress, Chl biosynthesis is weakened but Chl degradation is accelerated; hence, leaf chlorosis becomes one of the most obvious symptoms of leaf senescence [27]. Chl a and Chl b are involved in the absorption and transmission of light energy; however, Chl a is more sensitive to ROS than Chl b and degrades more rapidly in plants exposed to stressed conditions. It has been reported that sensitive bentgrass genotypes have shown greater decreases in total Chl and Chl a content and lower Chl a/Chl b ratios when compared to tolerant genotypes under heat stress [3], which is consistent with our experimental results. Senescence-associated genes (*AsSAG12, AsSAG39, Asl20*, and *Ash36*) have also indicated that CTS application could significantly alleviate heat-induced leaf senescence in creeping bentgrass. The Fv/Fm reflects the main photochemical efficiency of PSII and PIABS synthetically indicates the maximum photochemical efficiency of PSII, with the total number of activated reactive centers functioning as a sensitive photosynthetic indicator of photosynthetic performance, as well as for the health status of photosynthetic organs in plants under abiotic stress [28,29]. Some studies have shown that heat stress leads to a significant decrease in the Fv/Fm and PIABS of wheat, white clover, and creeping bentgrass [3,30,31]. Our results showed that Fv/Fm and PIABS decreased significantly under heat stress in the leaves of creeping bentgrass, and that exogenous CTS could mitigate the adverse effect of heat stress on the Fv/Fm and PIABS of creeping bentgrass.

In order to further reveal the effect of exogenous CTS on Chl metabolism in creeping bentgrass, the expression levels of key genes (*AsCHLH*, *AsPBGD*, *AsPPH*) involved in Chl biosynthesis and degradation were analyzed under normal conditions and heat stress. PBGD and CHLH are two key enzymes regulating Chl biosynthesis. PBGD can combine four porphobilinogen subunits to form a porphyrin ring, while CHLH is responsible for the insertion of magnesium ions into the porphyrin ring [32]. Chl anabolism is easily altered by a variety of abiotic stresses. It has been found that temperature-related stresses, including heat or cold stress, generated a decrease in PBGD and CHLH activities in cucumber (*Cucumis sativus*), thereby inhibiting Chl anabolism [33]. PPH catalyzes pheophytin into pheophorbide, which is the main process regulating Chl degradation [34,35]. The Chl degradation process is positively related to leaf senescence. Previous studies have shown that leaf senescence induced by submergence stress was associated with increased PPH activity and *PPH* gene expression in perennial ryegrass [36]. In contrast, reducing *PPH* gene expression significantly reduced leaf senescence in perennial ryegrass [37]. The decrease in Chl content in creeping bentgrass under heat stress is mainly due to the accelerated degradation of Chl [3,38]. Our current study demonstrates that the foliar application of CTS effectively alleviates the heat-induced decrease in Chl content. This could be related to the fact that CTS has been proven to significantly increase *AsPBGD* expression, as well as effectively alleviate heat-induced declines in *AsCHLH* expression while increasing the *AsPPH* transcript levels. Our previous studies found that mannose-regulated leaf senescence was not correlated with *PBGD* expression in white clover under water stress [39], but spermidine significantly up-regulated the *PBGD* expression, contributing to less Chl degradation occurring in the leaves of white clover during heat stress [31]. These findings indicate that different exogenous additives cause different effects on Chl metabolism. The CTS significantly mitigated heat-induced Chl degradation by enhancing the expression level of *AsPBGD*. It also alleviated heat-induced declines in *AsCHLH* expression, and increased *AsPPH* expression, resulting in ameliorated photosynthetic maintenance and reduced leaf senescence in creeping bentgrass under high temperature stress. Heat-induced Chl degradation and leaf senescence were also associated with the accumulation of reactive oxygen species (ROS). ROS (O_2_^–^ or H_2_O_2_) is a by-product of various cellular redox processes [40]. Under normal conditions, the ROS content in plants is in dynamic equilibrium. Under abiotic stresses, ROS metabolism and homeostasis is disturbed due to the imbalance in the capacity of cellular ROS scavenging [41]. Excessive ROS accumulation causes oxidative damage to cell membranes, proteins, and DNAs, resulting in programmed cell death and accelerated leaf senescence [42]. Senescence is controlled by the developmental age of plants, as well as abiotic stresses. It is a complex process involving many aspects of plant epigenetics, metabolic changes, and gene expression [40,43,44]. Increased production of ROS is a characteristic of senescent cells and an early response of plant cells to abiotic stress [43,45]. ROS also plays an important role by signaling molecules in the induction of senescence-related degradation processes [40]. The proline dehydrogenase gene (*PRODH*) promoted cellular senescence in a p53-dependent manner through the production of ROS; however, ROS scavengers could eliminate senescence induced by *PRODH* overexpression [46]. The accumulation of ROS such as O_2_^–^ or H_2_O_2_ was positively related to the accelerated senescence of mung bean (*Vigna radiata*) cotyledons [40]. The enhanced scavenging of ROS regulated by exogenous melatonin could significantly inhibit dark-induced leaf senescence in perennial ryegrass [47]. In the current study, the CTS application significantly decreased the heat-induced accumulation of ROS (O_2_^–^ and H_2_O_2_) or MDA. MDA is a membrane lipid peroxidation product produced by plants under adverse environmental conditions and is widely used as an indicator of oxidative damage in plants. This indicated that the CTS-regulated decrease in leaf senescence could be associated with improved ROS scavenging in creeping bentgrass under heat stress.

To mitigate oxidative damage and leaf senescence, the activation and enhancement of antioxidant defense system to eliminate excess ROS is an important strategy for plants to cope with stressful environments [48]. SOD converts O_2_^–^ into H_2_O_2_, while CAT, POD, and APX are mainly involved in H_2_O_2_ scavenging in plants [49]. The CTS has been reported to scavenge O_2_^–^, possibly due to the presence of a large number of hydroxyl and amino groups in its structure, which can react with ROS [50,51,52,53]. Exogenous CTS pretreatment attenuated salt-induced oxidative damage associated with significant increases in SOD, POD, CAT, and APX activities in rice, maize, and ajwain (*Carum copticum*) [54,55,56]. The application of CTS also enhanced the antioxidant defense system and mitigated oxidative damage in apple, common bean (*Phaseolus vulgaris*), and potato under drought or polluted water condition [14,57,58]. A recent field study revealed that foliar spray with CTS enhanced the tolerance of cucumber to high temperatures above 45 °C in summer through activating SOD, POD, and CAT activities, thereby reducing lipid peroxidation under heat stress [59]. Similar results were found in our study. The CTS application further increased the heat-induced activities of antioxidant enzymes (SOD, CAT, POD, and APX) and significantly reduced the ROS levels and MDA content, suggesting that the CTS attenuated heat-induced leaf senescence via improvement in the antioxidant defense system in creeping bentgrass.

In response to heat stress, plants have developed complex regulatory networks over a long evolutionary period. HSFs are thought to play central roles in transcriptional networks involved in the regulation of heat-responsive genes. HSFs in *Arabidopsis thaliana* are classified into three groups: HSFA, HSFB, and HSFC [60]. Transcriptomics revealed that high-temperature stress induced the upregulation of more than 65% of HSF-related genes, including *HSFA2, HSFB1*, and *HSFB2a* in *Arabidopsis* [61]. Previous studies have shown that *HSFA* plays a predominant regulatory role in *Arabidopsis* under heat stress, and that the upregulation of heat responsive genes could be eliminated in *hsfa1a/b/d/e* quadruple mutants [61,62]. *HSFA6b* mediated the response to ABA and heat tolerance in *Arabidopsis* [63]. The overexpression of *FaHSFA2a* significantly reduced heat-induced cell membrane damage and leaf senescence in tall fescue (*Festuca arundinacea*) [64]. Heat shock response in plants is associated with the increased expression of heat shock proteins (HSPs) in an HSF-dependent manner [63,65]. Abiotic stress could destroy protein structure and function, resulting in plant senescence. Thus, protein degradation is one of the basic features of plant senescence. HSPs exhibit a molecular chaperone role to enhance plant resistance to heat stress by stabilizing proteins and ensuring the proper assembly and folding of proteins under heat stress [66]. The overexpression of *PIHSP70* conferred a greater tolerance to heat stress in herbaceous peony (*Paeonia lactiflora*) by maintaining better photosynthesis and mitigating oxidative damage [67]. The application of exogenous GABA enhanced transcript levels of *HSP* genes including *HSP70* and *HSP82*, thereby maintaining water and metabolic balance in creeping bentgrass under heat stress [68]. Up to now, the effect of CTS on regulating the HSF pathway for heat tolerance has not been well-elucidated in plants. The transcriptional analysis in the current study found that heat stress significantly increased the transcript levels of *AsHSFA-6a* and *AsHSP82*, while exogenous CTS treatment further increased the expression levels of these two genes during heat stress. These findings suggest that the CTS mitigated heat-induced damage and senescence in the leaves of creeping bentgrass in relation to the HSF pathway, but that the potential mechanisms involved need to be further investigated in our future study.

## 4. Materials and Methods

### 4.1. Plant Material and Treatment

Creeping bentgrass (cv. Penn A-4) was used as an experimental material. Seeds were sterilized with 0.1% potassium permanganate solution for 10 min and then washed three times with distilled water. The sterilized seeds (8 g/m^2^) were evenly sowed in containers (23 cm length, 15 cm width, 10 cm height) containing quartz sand and appropriate distilled water. All the containers were randomly placed in growth chambers (23/18 °C (day/night), 65% relative humidity, and 700 µmol m^−2^·s^−1^ PAR) for 10 days of germination. Later, seedlings were cultivated in Hoagland’s solution [69] for another 23 days. During this period of cultivation, the Hoagland’s solution was renewed at intervals of every two days to avoid the changes in the pH value and solution concentration.

For foliar CTS pretreatment, plants were sprayed twice a day with 10 mL of 0.1 g L^−1^ CTS (Sigma-Aldrich, Saint Louis, MO, USA) solution or distilled water (Control) for 3 days before being exposed to heat stress. For heat stress treatment, the CTS-pretreated and untreated plants were grown in a high-temperature growth chamber (38/33 °C (day/night), 65% relative humidity, and 700 µmol m^−2^·s^−1^ PAR) for 15 days. Non-stressed controls (the CTS-pretreated and unpretreated plants) were grown under optimal temperature conditions at 23/18 °C (day/night), 65% relative humidity, and 700 µmol m^−2^·s^−1^ PAR for 15 days. A total of four treatments, including C (the CTS-untreated control plants grown under normal condition), CTS (the CTS-pretreated control plants grown under normal condition), H (the CTS-untreated plants grown under heat stress), and H + CTS (the CTS-pretreated plants grown under heat stress), were utilized for the experiment. The plastic containers were laid out in growth chambers under a completely randomized design (CRD) with four independent biological replicates for each treatment. Leaf samples were collected after 15 days of heat stress and utilized for subsequent physiological and gene expression analyses.

### 4.2. Determination of Water Status and Photosynthetic Parameters

The RWC was determined by the drying method [70]. OP was estimated by following Blum’s method [71]. Chl content was measured by using the acetone-ethanol mixture method [72]. The Fv/Fm and PIABS were recorded with a Chl fluorescence system (Pocket PEA, Hansatech Instruments Ltd., King’s Lynn, UK).

### 4.3. Determination of Oxidative Damage and Antioxidant Enzyme Activities

O_2_^–^ was determined using the method of Elstner [73]. H_2_O_2_ was measured by following the procedure of Velikova [74]. MDA content was estimated by the thiobarbituric acid method [75]. EL was determined by the conventional conductivity meter method [76]. SOD activity was estimated using the riboflavin-NBT method [77]. CAT activity was measured using the UV absorption method [78]. POD activity was determined using the guaiacol colorimetric method [78]. APX activity was estimated using the protocols of Nakano and Asada [79].

### 4.4. Genes Expression Analyses

Gene transcription levels were detected by real-time quantitative polymerase chain reaction (qRT-PCR). Total RNA was extracted from 0.1 g of fresh leaves using the RNAprep Pure Plant Kit (Tiangen, China). The RNA was reverse-transcribed to the cDNA using the PrimeScript™ RT reagent Kit with gDNA Eraser (TaKaRa, Japan). Primer sequences of Chl synthesis genes (*AsCHLH*, *Mg-chelatase*; *AsPBGD*, *porphobilinogen deaminase*), a Chl degradation gene (*AsPPH*, *pheophytinase*), a heat shock factor gene (*AsHSFA-6a*), a heat shock protein gene (*AsHSP82*), and senescence-associated genes (*AsSAG12*, *AsSAG39*, *Asl20*, *Ash36*) are shown in Table 1 [3]. The PCR protocol conditions for all genes were as follows; 5 min at 94 °C and 30 s at 95 °C (40 repeats of denaturation), annealing 45 s at 58–64 °C, and amplification from 60 to 95 °C. The transcript levels of all genes were calculated according to the formula 2^−∆∆Ct^ [80].

### 4.5. Statistical Analysis

The data were analyzed by one-way ANOVA using SPSS 20 (IBM, Armonk, NY, USA). The significant differences among treatments were tested based on the least significant difference (LSD) at *p* ≤ 0.05.

## 5. Conclusions

Under high-temperature conditions, detrimental effects including declines in RWC and photochemical efficiency and significant increases in ROS accumulation, membrane lipid peroxidation, and Chl degradation were detected in the leaves of creeping bentgrass. The exogenous application of an optimal dose of CTS significantly alleviated heat-induced stress damage to creeping bentgrass associated with an improvement in water balance, ROS scavenging, and the maintenance of Chl metabolism and photosynthesis. In response to heat stress, CTS-treated creeping bentgrass significantly enhanced the transcript levels of genes involved in Chl biosynthesis (*AsPBGD* and *AsCHLH*) and reduced the expression level of a Chl degradation-related gene (*AsPPH*), as well as senescence-associated genes (*AsSAG12*, *AsSAG39*, *Asl20*, and *Ash36*) compared with untreated plants, thus reducing leaf senescence and ameliorating photosynthetic maintenance. In addition, the foliar spray of CTS activated antioxidant enzyme activities (SOD, CAT, POD, and APX), thereby effectively mitigating heat-induced oxidative damage and leaf senescence. Heat tolerance regulated by the CTS in creeping bentgrass was also associated with the improved transcription of *AsHSFA-6a* and *AsHSP82*, but the mechanism involved still needs to be further studied.

## Figures and Tables

**Table 1:** Primer sequences and annealing temperature of the analyzed genes.

Target Gene	Forward Primer (5′–3′)	Reverse Primer (5′–3′)	Tm (°C)
*β-Actin*	CCTTTTCCAGCCATCTTTCA	GAGGTCCTTCCTGATATCCA	58
*AsPBGD*	TAGCGCTGCGGATTAGAACT	GAAGGATAACGAACCGCTGA	55
*AsCHLH*	CATCAGGGCGGATAGAGAGA	TCTGCCACAATCAGCTTCAG	56
*AsPPH*	GAATGTCATTGCCGTCTGAA	CAATGAAATGCTGGACCTGA	53
*AsHSFA-6a*	CACCTTCGAGGAGCTGGCATTG	TGTCTATCTCCGCCTGCTCATCC	62
*AsHSP82*	GAGCCTGACGGACAAGAGCAAG	GGAGTGAAGCAGAGTAACGAGACG	64
*AsSAG12*	CCCAGCAGTTTACTGGCTTT	AAGCAGGTGCCTTGAAACTT	54
*AsSAG39*	CCTCGCTGTTCTTGCCGTGAG	CGTGCTCAGCCATCCACTTCTC	63
*Asl20*	GGGTAGACGGCAACGATACT	TACTTGGTTGAATCGTCGGA	60
*Ash36*	TGGGAATGTGTTCAGGGTAA	TCACCTCGATGAGGTAGTCG	55

## Data Availability

Not applicable.

## Sample Availability

Not available.
